# Speciation analysis of fungi by liquid atmospheric pressure MALDI mass spectrometry

**DOI:** 10.1007/s00216-025-06094-6

**Published:** 2025-09-05

**Authors:** Lily R. Adair, Ian M. Jones, Rainer Cramer

**Affiliations:** 1 Department of Chemistry, School of Chemistry, Food and Pharmacy, University of Reading, Reading, RG6 6DX UK; 2 School of Biological Sciences, University of Reading, Reading, RG6 6AJ UK

**Keywords:** Biotyping, Fungi, LAP-MALDI, MALDI, Mass spectrometry, Speciation

## Abstract

Fungal pathogens pose a growing threat to global health, necessitating rapid and accurate identification methods. Here, liquid atmospheric pressure matrix-assisted laser desorption/ionisation (LAP-MALDI) mass spectrometry (MS) is applied to fast lipid and protein profiling of *Candida albicans* and *Saccharomyces cerevisiae* from cultured colonies. Species-specific lipid profiles were observed in the *m/z* 600–1100 range, dominated by phospholipids as confirmed by tandem mass spectrometry (MS/MS). Following simple solid phase extraction clean-up, LAP-MALDI mass spectra revealed multiply charged protein ions suitable for MS/MS analysis. For *C. albicans*, the fully mature, species-specific WHS11 protein (~ 7 kDa; P43074) was detected intact and confidently identified by top-down MS/MS proteoform sequencing, including the cleavage of the N-terminal methionine initiator and the associated N-terminal acetylation. For *S. cerevisiae*, a set of proteoforms were sequenced by MS/MS analysis, which led to the identification of two species-specific proteins within the ‘UniProtKB reference proteomes + Swiss-Prot’ target database. One of these was also detected intact, and sequenced and identified as the fully mature HSP12 protein (~ 11.5 kDa; P22943). This work demonstrates the potential of LAP-MALDI MS and MS/MS biotyping as a powerful, label-free platform for rapid fungal classification and proteoform characterisation, offering substantial improvements over conventional MALDI biotyping.

**Graphical Abstract:**

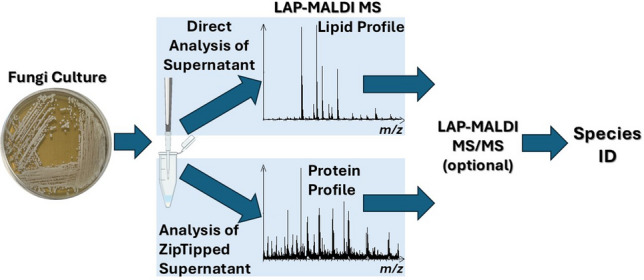

## Introduction

The kingdom *Fungi* encompasses a broad range of organisms, from simple unicellular species such as yeasts to complex multicellular forms like molds and mushrooms. These organisms play critical roles in both ecological systems and industrial applications. Yeasts, particularly *Saccharomyces cerevisiae*, have long been integral in food and beverage fermentation, biofuel production, and the synthesis of pharmaceuticals and fine chemicals. *S. cerevisiae* was the first eukaryotic organism to have its genome fully sequenced and remains a widely used model in molecular biology and biotechnology [1]. Other fungi such as arbuscular mycorrhizal fungi (AMF) are key to sustainable agriculture, functioning as plant biofertilizers, promoting plant nutrient uptake and improving soil health [2].

The fungi also include important pathogens. In both clinical and environmental contexts, fungal infections pose a serious threat to both human and animal health, often requiring prolonged treatment and exhibiting limited responsiveness to standard antimicrobial therapies. Invasive fungal infections contribute to approximately 3.8 million deaths annually, with fungi being the primary cause in about 2.5 million of these cases, underscoring their significant impact on global mortality [3]. In 2022, the World Health Organization (WHO) published its first Fungal Priority Pathogens List (FPPL), highlighting 19 fungal species that pose the greatest threat to public health, including *Candida auris*, *Aspergillus fumigatus*, and *Cryptococcus neoformans* [4]. These pathogens contribute to high morbidity and mortality, particularly among immunocompromised individuals, and are increasingly resistant to existing antifungal treatments.

Given that delayed or inappropriate antifungal therapy is strongly associated with high mortality rates, the rapid and accurate identification of fungal pathogens is critical for guiding effective treatment [5]. Beyond clinical care, efficient fungal identification is also critical in food safety, pharmaceutical production, environmental surveillance, and agriculture. The increasing emergence of antifungal-resistant and atypical strains has increased demand for fast, cost-effective, and accurate methods to distinguish fungal species in these varied contexts [6].

Traditionally, fungal identification in clinical laboratories has relied on phenotypic techniques such as microscopy [7], culture-based morphology [8, 9], and biochemical assays [8]. These methods, including germ tube tests and carbohydrate assimilation panels (e.g., API 20 C AUX, Vitek 2), are low-cost and widely accessible but typically require 1–3 days to produce results [10–12]. They are further limited by the variability in growth conditions, phenotypic overlap between species, and reliance on expert interpretation, often resulting in misidentifications, particularly among closely related species like *Candida albicans* and *C. dubliniensis* [13]. Mold identification using these methodologies is particularly challenging due to their sporulation potential and diverse morphologies, which require subjective visual assessment.

Molecular diagnostic approaches such as real-time PCR, rDNA sequencing, restriction fragment length polymorphism (RFLP), and fluorescence in situ hybridization (FISH) offer greater specificity but are time-consuming, labour-intensive, and often require specialised equipment and expertise not universally available in routine settings [14–17].

Matrix-assisted laser desorption/ionisation (MALDI) time-of-flight (TOF) mass spectrometry (MS) has transformed microbial biotyping and is now widely regarded as the gold standard for bacterial identification. Its application in fungal diagnostics, especially for yeasts, is expanding rapidly, with growing use for filamentous fungi [18–22]. By generating species-specific proteoform profiles and comparing them to reference libraries, MALDI-TOF biotyping enables rapid, high-throughput, and cost-effective microbial identification, often within minutes, markedly faster than conventional phenotypic or molecular approaches.

However, a major limitation of conventional (solid-phase) MALDI as currently used in clinical MALDI-TOF biotyping is the generation of predominantly singly charged ions, which restricts peptide and proteoform fragmentation efficiency and limits compatibility with high-resolution tandem mass spectrometry platforms such as Q-TOF and orbitrap instruments. In contrast, electrospray ionisation (ESI) generates abundant peptide/protein ions with a higher number of charges, that are more amenable to downstream MS/MS analysis resulting in superior fragmentation and lower *m/z* values that allow the employment of high-performing Q-TOF and orbitrap instruments. Furthermore, the limited scope of MALDI-TOF MS profile databases, which often lack comprehensive coverage of diverse or newly described species, can lead to misidentifications or failure to identify organisms, particularly those underrepresented in commercial libraries, and contributes to challenges in distinguishing closely related species. Compounding this challenge, the chitin-rich cell walls of fungi hinder protein extraction, often resulting in suboptimal mass spectra [23].

To overcome these barriers, novel strategies have been explored to improve fungal cell lysis and protein detection. Electroporation techniques, which apply high-voltage pulses to permeabilise fungal membranes directly on solid media, have been coupled with ambient ionisation strategies such as liquid extraction surface analysis (LESA) to enable in situ fungal protein profiling [24]. Chemical approaches using membrane-disrupting agents, such as the cationic amphiphilic dendron C18-G1, have similarly enhanced mass spectral quality by improving protein yield when directly applied to MALDI samples that are already spotted on sample plates [25]. Other emerging ambient ionisation methods like paper spray mass spectrometry (PS-MS) have shown promise in rapidly characterising microbial lipids and proteins with minimal sample preparation [26, 27].

Liquid Atmospheric Pressure-MALDI (LAP-MALDI), a newer ambient ionisation technique developed and optimised over the past decade, addresses many limitations of traditional MALDI, demonstrating significant success in MS biotyping and identification of clinically relevant bacteria and in MS-based veterinary diagnostics [28, 29]. LAP-MALDI facilitates the generation of ESI-like multiply charged ions, allowing the use of high-performing HRMS instruments and therefore the effective use of MALDI for MS(/MS) analysis. The liquid nature of the MALDI sample also promotes a stable ion yield while consuming minimal sample volume (picolitres).

In this study, we extend the use of LAP-MALDI to fungal diagnostics, demonstrating its potential for rapid and accurate identification of yeast species. By simultaneously analysing lipid and protein profiles in a single LAP-MALDI mass spectrum, this approach enables species-level classification, with the added capacity for top-down MS/MS proteoform sequencing, facilitating detailed pathogen characterisation without the need for machine learning-based predictive classification models. We apply a straightforward ethanol extraction protocol to *Candida albicans* and *Saccharomyces cerevisiae* isolates, two species that represent medically important and model organisms, respectively. The findings of this study demonstrate the potential of LAP-MALDI as a powerful tool for fungal identification and characterisation.

## Material and methods

### Reagents and materials

*Candida albicans* (NCPF 3179) was obtained in freeze-dried format from the National Collection of Pathogenic Fungi (NCPF), Culture Collections, UK Health Security Agency (Salisbury, UK). *Saccharomyces cerevisiae* was sourced from commercially available baker’s yeast (dried sachet for breadmaking).

α-Cyano-4-hydroxycinnamic acid (CHCA) was purchased from Bruker Daltonics (Coventry, UK). Trifluoroacetic acid (TFA), acetone, and ethanol (≥ 99% purity, laboratory reagent grade) were obtained from Fisher Scientific (Loughborough, UK). LC–MS-grade water and acetonitrile (ACN) were purchased from Honeywell (Bracknell, UK). Peptone and bacteriological agar were purchased from Thermo Fisher Scientific (Loughborough, UK). Formic acid, propylene glycol (PG), yeast extract, and D-(+)-glucose were obtained from Sigma-Aldrich (Gillingham, UK).

### Sample preparation

*C. albicans* cultures were revived from freeze-dried stocks following the supplier’s instructions while *S. cerevisiae* was rehydrated in sterile phosphate-buffered saline for 10 min.

The yeast extract–peptone–glycerol (YPG) culture medium was prepared using 1% w/v yeast extract, 2% w/v peptone, 2% w/v glucose, and 2% w/v agar. The pH was adjusted to a value of 6.5 using hydrochloric acid (HCl) prior to autoclaving the YPG medium at 121 °C for 22 min to ensure sterility.

Both *Candida albicans* and *Saccharomyces cerevisiae* were grown on the solidified YPG medium as prepared above and incubated at room temperature for 24–72 h under aerobic conditions.

After growth, approximately 5 μL of fungal biomass was suspended in 30 μL of sterile water, followed by the addition of 90 μL of ethanol. The suspension was vortexed and centrifuged at 12,000 *g* for 2 min. The supernatant was discarded, and the sample was centrifuged again for 1 min. The remaining supernatant was carefully removed, and the pellet was air-dried at room temperature for 5 min. The dried pellet was resuspended in 20 μL of 70% formic acid, mixed thoroughly, and incubated at room temperature for 5 min. Subsequently, 20 μL of acetonitrile was added, the sample was vortexed and centrifuged at 12,000 *g* for 2 min. The residual pellet was washed with acetone, resuspended in 50 μL of 0.1% TFA, and centrifuged again at 12,000 *g* for 2 min. An aliquot of 10 μL of the resulting supernatant underwent sample clean-up using C18 ZipTips (ZTC18S096; EMD Millipore, Merck, Gillingham, UK), following the manufacturer’s protocol. Proteins were eluted from the ZipTips using 10 μL of a 50:50 (v/v) acetonitrile/water solution, and the eluted material was used for subsequent protein analysis. The remainder of the supernatant that did not undergo ZipTip clean-up was used for lipid profile analysis.

### LAP-MALDI sample droplet preparation

To prepare the LAP-MALDI matrix, CHCA was dissolved in a 70:30 (v/v) ACN:water solution to a concentration of 15 mg/mL. The CHCA solution was vortexed thoroughly, after which PG was added using a volume of PG that was 70% of the volume of the CHCA solution, followed by additional vortexing to ensure homogeneity. All LAP-MALDI samples were prepared by dispensing 500 nL of the matrix solution onto a stainless-steel MALDI sample plate, which was mixed on-plate with 500 nL of fungal extract via repeated aspiration and dispensing, forming a homogeneous 1-μL droplet per sample.

### LAP-MALDI MS and MS/MS

LAP-MALDI MS analyses were conducted using a modified SYNAPT™ G2-Si Q-TOF mass spectrometer (Waters Corporation, Wilmslow, UK) equipped with a custom-built atmospheric pressure MALDI source, as previously described [30]. In brief, a stainless steel 384-well sample plate was positioned orthogonally at a distance of 3 mm from a heated ion transfer tube, with a nitrogen counter-gas flow of 210 L/h to aid ion transmission. Samples were irradiated using a 343-nm diode-pumped solid-state Yb:YAG laser (FlareNX 343–0.2–2; Coherent, Santa Clara, USA) operating at a pulse repetition rate of 50 Hz. The laser beam was directed at a 30° angle relative to the sample plate normal and focused onto the centre of each sample droplet, delivering approximately 10 μJ per 3-ns pulse to facilitate desorption. All data were acquired in positive ion mode with the instrument operating in ‘Mobility TOF’ and sensitivity mode. Although ‘Mobility TOF’ mode was used, ion mobility was not exploited in this study and ‘TOF’ mode should therefore also be adequate. The ion source was operated with an extraction potential of approximately 3 kV. Instrument calibration was performed manually across an *m/z* range of 100–2000 using 500 ng/μL caesium iodide dissolved in isopropanol:water (1:1, v/v), mixed 1:1 (v/v) with the matrix solvent system (excluding CHCA), and processed using Intellistart software (MassLynx 4.2; Waters).

For tandem MS (MS/MS) experiments, precursor ions were selected using quadrupole isolation, with low-mass (LM) and high-mass (HM) resolution values set at 4.8 and 15, respectively. Collision-induced dissociation (CID) was performed in the trap cell using collision energies ranging from 25 to 60 V, depending on the *m/z* value and charge state of the selected precursor. Multiple charge states were sequentially isolated and fragmented to generate detailed MS/MS spectra. Data for all acquisitions were acquired across an *m/z* range of 50–2000.

### Lipid MS data analysis

For both species, the top ten most intense centroided monoisotopic *m/z* values within the range of 600–1100 were selected and searched using the LIPID MAPS search routine and database (https://www.lipidmaps.org/bulk_search; last updated: 2025–08-18). The *m/z* values were queried using a tolerance of *m/z* (±) 0.005, against all quasi-molecular positive ions and all lipid classes.

### Proteoform MS/MS data analysis

Raw mass spectral data were processed using Mascot Distiller (version 2.8.5.1, 64-bit; Matrix Science, London, UK), which performed automated peak picking. For the ‘MS Peak Picking’ module, a correlation threshold (Rho) of 0.7 and a minimum signal-to-noise ratio (S/N) of 3 were applied. Baseline correction was carried out using the isotope distribution fit method, allowing up to 500 peak iterations per scan. Peak width parameters were configured with a minimum of 0.005 Da, an expected width of 0.05 Da, and a maximum width of 0.5 Da. The resulting exported peak list consisted of the monoisotopic masses of singly charged fragment ion equivalents.

MS/MS data were further processed following a previously established method [31]. Briefly, fragment ions from each MS/MS acquisition, derived from the same charge state distribution, were grouped within an *m/z* tolerance of ± 0.1. Peaks were filtered by applying a minimum intensity threshold of 10 and were required to be present in the MS/MS spectra of at least two distinct charge states. The curated peak list was then submitted to the Mascot MS/MS Ions Search tool (version 3.1; Matrix Science), with database searches conducted against both the Mascot contaminants database (downloaded on 20 January 2025; 247 sequences; 128,130 residues) and the Swiss-Prot database (downloaded on 22 May 2024; 571,282 sequences; 206,678,396 residues). Search parameters included monoisotopic mass values, a precursor ion mass tolerance of 50 ppm, and a fragment ion tolerance of 0.2 Da. The instrument type was set to ‘MALDI-QIT-TOF’, with ‘Acetyl (Protein N-term)’ selected as a variable modification. ‘None’ was selected for enzyme and ‘All entries’ for taxonomy.

BLAST searches were undertaken with the Mascot-identified amino acid sequences submitted to the search routine at www.uniprot.org/blast, using the default parameters with ‘UniProtKB reference proteomes + Swiss-Prot’ as target database.

Charge state deconvolution was achieved by using UniDec (version 7.0.0b; https://github.com/michaelmarty/UniDec/releases) [32]. The *m/z* range was set to 500–2000, with background subtraction applied. The charge range was defined as 2–20, and the mass range was set to 2,000–8,000 Da for *C. albicans* and 2,000–14,000 Da for *S. cerevisiae*. Isotopic distributions were set to monoisotopic during deconvolution. All other parameters were left at their default settings.

## Results

**Fig. 1:**
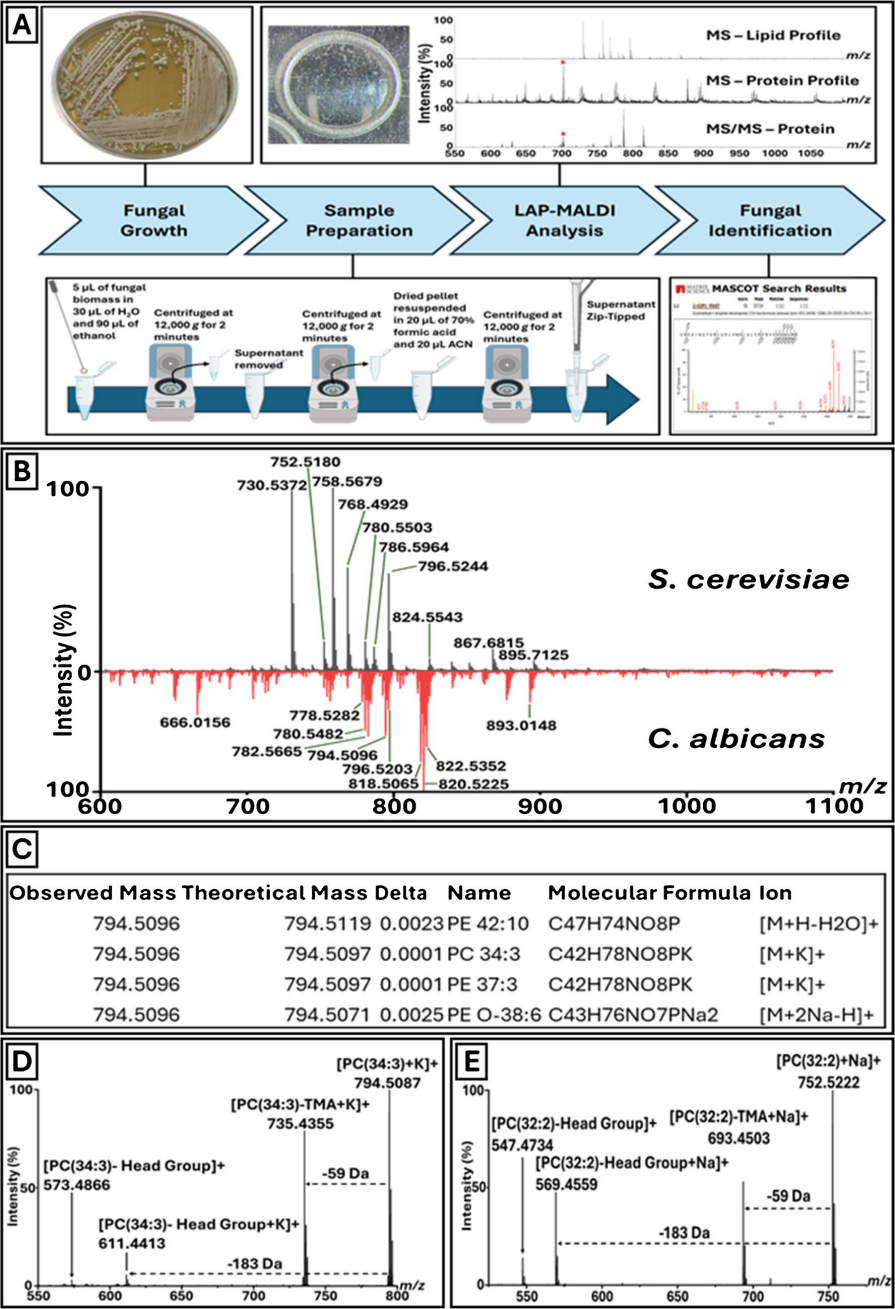
LAP-MALDI MS and MS/MS Analysis of Fungal Cultures. **A** Schematic overview of the workflow from fungal culturing and sample preparation to LAP-MALDI MS and MS/MS data acquisition and analysis. **B** Representative LAP-MALDI MS profile spectra of lipids from *S. cerevisiae* (black) and *C. albicans* (red). **C** LIPID MAPS matches for the monoisotopic ion signal at *m/z* 794.5096 of a *C. albicans* lipid. **D** MS/MS spectrum of the MS profile precursor ion signal at *m/z* 794.5096. **E** MS/MS spectrum of the *S. cerevisiae* MS profile precursor ion signal at *m/z* 752.5180

Two fungal species were studied in a first-of-its-kind investigation into the application of LAP-MALDI MS for fungal characterisation. Colonies of *S. cerevisiae* and *C. albicans* were subjected to a simple, routine biotyping extraction protocol, enabling lipid and protein profiling within 30 min from harvest to LAP-MALDI MS data acquisition and analysis (Fig. 1A).

Notably, although protein signals were observable in the original supernatant prior to ZipTip clean-up, the clean-up step was performed to remove lipids and improve protein recovery, thereby increasing signal intensity. Without ZipTip clean-up, the sample extracts of both microorganisms exhibited highly distinctive lipid profiles in the *m/z* range of approximately 600–1100, which enabled clear differentiation between species (Fig. 1B). However, many of the peaks observed in this region are common across multiple fungal species, albeit with varying relative abundances.

Putative identification of lipid ion peaks was conducted using the open-access LIPID MAPS Structure Database (Fig. 1C). The major lipid classes of the LAP-MALDI mass spectra assigned by LIPID MAPS searches (with a tolerance of *m/z* ± 0.005) included membrane phospholipids such as phosphatidylcholines (PCs), phosphatidylethanolamines (PEs), and phosphatidylinositols (PIs), along with their lyso forms (e.g., LPCs). Neutral storage lipids, diacylglycerol (DAG) and triacylglycerol (TAG) species, were also assigned, although less frequent. These exhibited a higher degree of variability between the fungal strains. In general, strong potassiated ion signals were obtained, most likely due to the use of phosphate-buffered saline.

As shown in the mass spectra of Fig. 1B, multiple lipid peaks are distinct between the two species. Some of these discriminative ions were further identified through MS/MS analysis and accurate mass measurement. For example, the lipid profile for *C. albicans* revealed a lipid ion signal at *m/z* 794.5096 that only led to four putative assignments of lipids, of which the two assignments with the smallest mass deviation from the measured mass were the potassiated PC 34:3 and PE 37:3 (see Fig. 1C). However, the MS/MS data of the same precursor ion (detected at *m/z* 794.5087 in the MS/MS spectrum; Fig. 1D) can be unambiguously assigned to the fragmentation of the potassium adduct ion of PC(34:3). This identification was supported by the characteristic neutral losses of ~ 59 Da, corresponding to the loss of trimethylamine (TMA), yielding a fragment ion peak at *m/z* 735.4355, and ~ 183 Da, corresponding to the loss of the PC headgroup, yielding a fragment ion peak at *m/z* 611.4413. The measured mass differences between the precursor ion and the two fragment ions are 59.0732 Da and 183.0674 Da, respectively, and are therefore within 10 ppm of the theoretical neutral mass losses of 59.0735 Da and 183.0660 Da. Further loss of ~ 38 Da in addition to the ~ 183 Da loss, resulting in a fragment ion at m/z 573.4866, indicates the loss of potassium. Strong ion signals at *m/z* 778.5282, corresponding to the sodiated PC(34:3) were also observed in the lipid MS profile.

Similarly, the *S. cerevisiae* precursor ion at *m/z* 752.5180 (detected at *m/z* 752.5222 in the MS/MS spectrum; Fig. 1E) can be assigned by MS/MS analysis to the sodiated PC(32:2) lipid, as evidenced by the loss of TMA resulting in a fragment ion at *m/z* 693.4503, and PC headgroup losses resulting in fragment ions at *m/z* 569.4559 and *m/z* 547.4734, corresponding to the singly sodiated and protonated forms, respectively. Strong ion signals at *m/z* 730.5406, corresponding to the protonated PC(32:2), and at *m/z* 768.4929, corresponding to the potassiated PC(32:2), were also observed in the lipid MS profile.

**Fig. 2:**
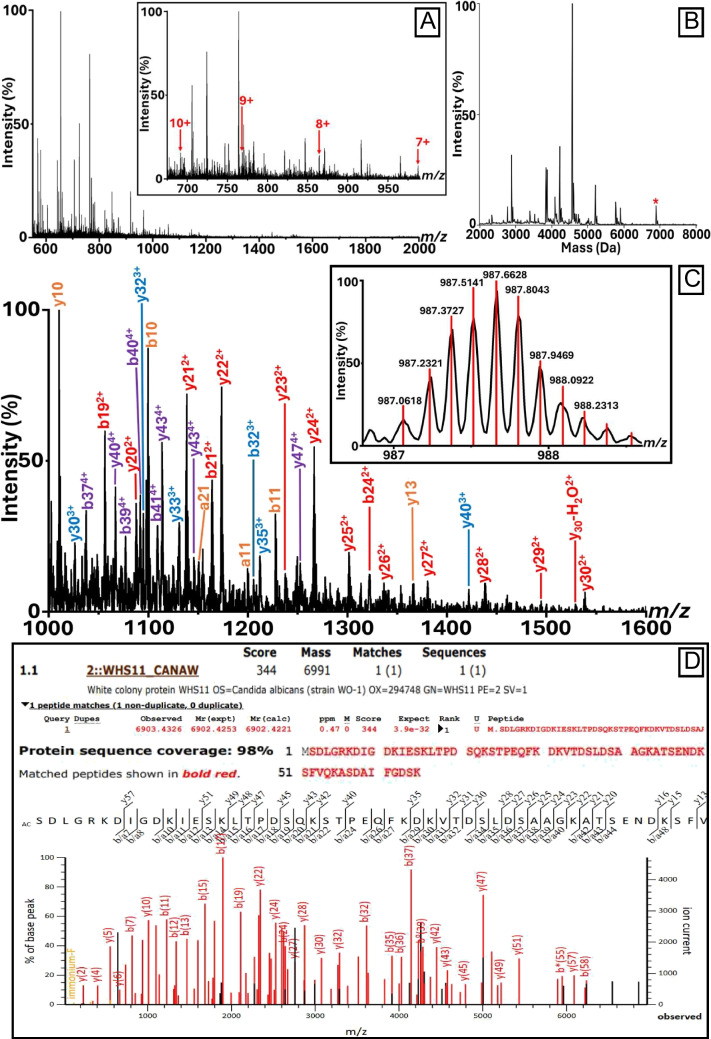
*C. albicans* Identification by LAP-MALDI MS/MS Sequencing. **A** LAP-MALDI MS profile of *C. albicans* following C18-ZipTip extraction. The inset shows the charge states used for MS/MS analysis. **B** Deconvoluted spectrum corresponding to the MS profile shown in panel **A**. The peak marked with an asterisk (*) was selected for CID fragmentation. **C** LAP-MALDI MS/MS spectrum of the proteoform (7 +) detected at *m/z* 987.06 (see panel **A**), enabling identification as the white colony protein WHS11. The inset displays the precursor ion signals with centroided isotopologues. **D** Mascot MS/MS Ions Search results from the MS/MS data shown in panel **C**, confirming a significant species-specific match to the *C. albicans* Swiss-Prot entry P43074

**Fig. 3:**
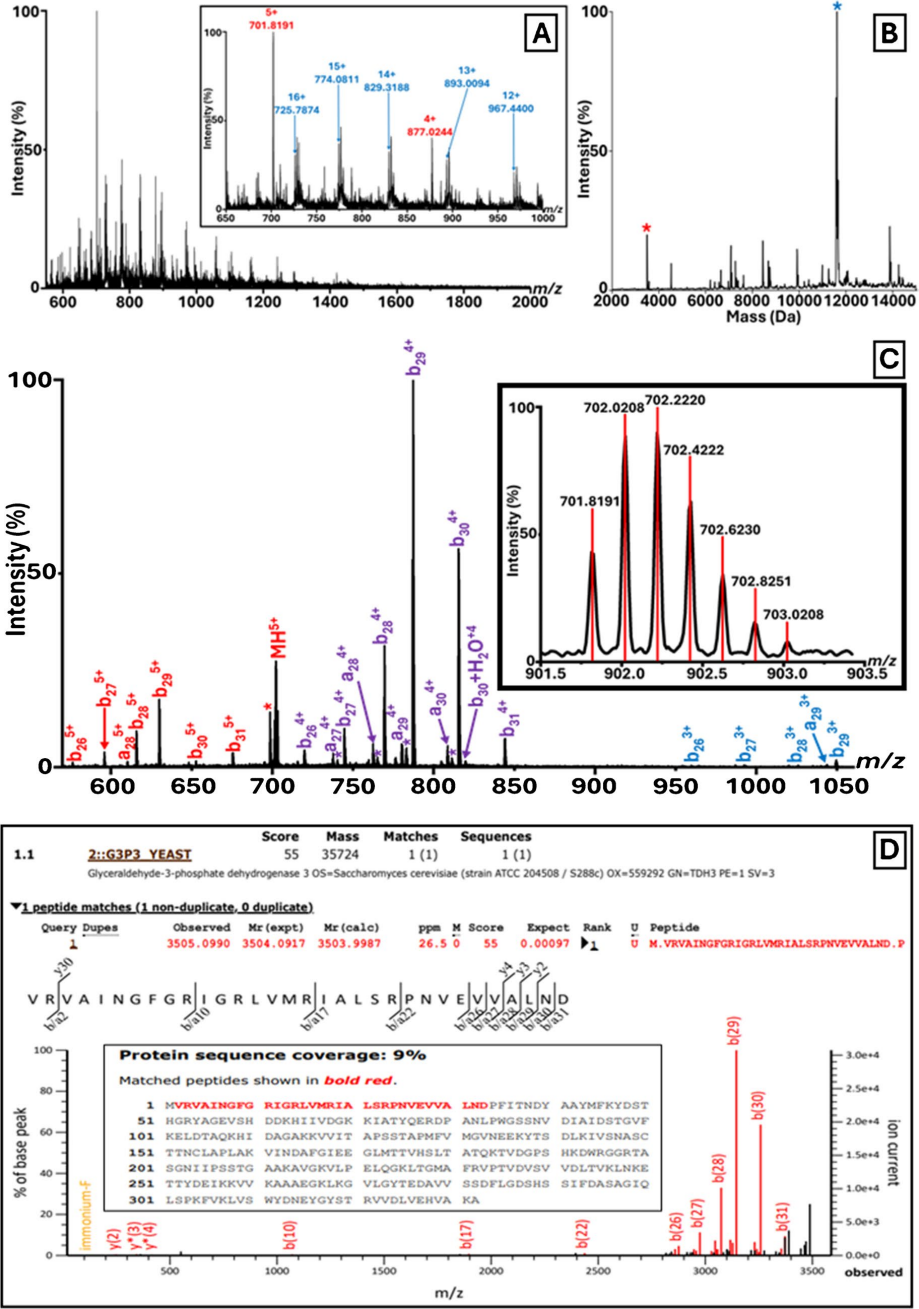
*S. cerevisiae* Identification by LAP-MALDI MS/MS Sequencing. **A** LAP-MALDI MS profile of *S. cerevisiae* following C18-ZipTip extraction. The inset shows the two charge state distributions used for MS/MS analysis. **B** Deconvoluted spectrum corresponding to the MS profile shown in panel **A**. The peaks marked with an asterisk (*) were selected for CID fragmentation. **C** LAP-MALDI MS/MS spectrum of the proteoform (5 +) detected at *m/z* 701.82 (see panel **A**), enabling identification as a fragment of glyceraldehyde-3-phosphate dehydrogenase 3. The inset displays the precursor ion signals with centroided isotopologues. **D** Mascot MS/MS Ions Search results from the MS/MS data shown in panel **C**, confirming a significant species-specific match to the *S. cerevisiae* Swiss-Prot entry P00359

Following ZipTip clean-up, a plethora of multiply charged ion signals corresponding to proteins were observed for both *C. albicans* and *S. cerevisiae* (Figs. 2 and 3, respectively). In the case of *C. albicans*, MS/MS analysis (Fig. 2C) of the ion signals highlighted in Fig. 2A (see inset) provided a significant search hit only to the white colony protein WHS11 (Swiss-Prot accession no. P43074; Fig. 2D) with a protein sequence coverage of 100% considering the fully mature protein after cleavage of the N-terminal methionine. A BLAST search of the identified amino acid sequence against the ‘UniProtKB reference proteomes + Swiss-Prot’ target database showed that this sequence is species-specific. The observed deconvoluted monoisotopic mass obtained by Mascot Distiller is 6902.43 Da and therefore approximately 89 Da lower than that of the theoretical mass obtained from the Swiss-Prot database entry. This discrepancy is attributed to the absence of the initiator methionine (−131 Da) and the presence of N-terminal acetylation (+42 Da), resulting in a mass difference of −89 Da, which is also reflected in the b-ion series of the MS/MS spectrum.

**Fig. 4:**
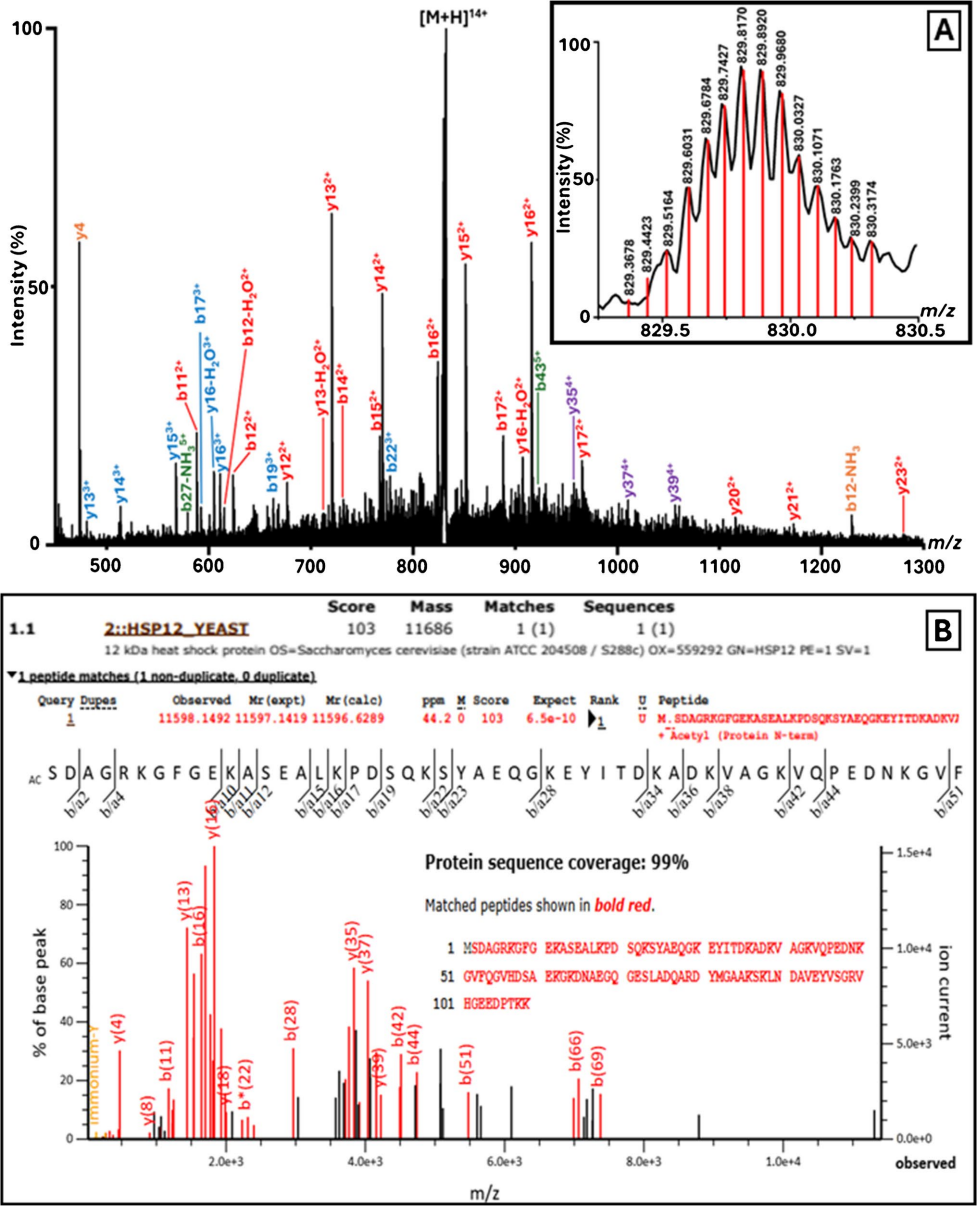
*S. cerevisiae* Identification by LAP-MALDI MS/MS Sequencing. **A** LAP-MALDI MS/MS spectrum of the proteoform (14 +) detected at *m/z* 829.37, enabling identification as 12-kDa heat shock protein HSP12. The inset displays the precursor ion signals with centroided isotopologues. **B** Mascot MS/MS Ions Search results from the MS/MS data shown in panel **A**, confirming a significant species-specific match to the *S. cerevisiae* Swiss-Prot entry P22943

For *S. cerevisiae*, the MS profile spectrum displays two major charge state distributions (Fig. 3A, see inset, and Fig. 3B). The ion signals detected at *m/z* 701.8191 (5 +) (Fig. 3C) and 877.0244 (4 +) were selected for CID fragmentation and identified as an approximately 3500 Da fragment of glyceraldehyde-3-phosphate dehydrogenase 3 (Swiss-Prot accession no. P00359; Fig. 3D). Another prominent charge state distribution with a deconvoluted mass of 11,596.17 Da (Fig. 3B) was also subjected to MS/MS analysis (see Fig. 4A for MS/MS spectrum of the precursor ion at *m/z* 829.3678) and identified as the 12-kDa heat shock protein HSP12 (Swiss-Prot accession no. P22943; Fig. 4B), which also lacked the initiator methionine and featured N-terminal acetylation. BLAST searches of these two identified amino acid sequences against the ‘UniProtKB reference proteomes + Swiss-Prot’ target database showed that both sequences are also species-specific.

## Discussion

The ability of LAP-MALDI MS to detect bacterial lipids, enabling species-level discrimination, has been previously demonstrated [33]. In this proof-of-concept study, we further extend LAP-MALDI’s application to fungal biotyping. Two fungal species, *Candida albicans* and *Saccharomyces cerevisiae*, were successfully differentiated based on their lipid and protein MS profiles using a rapid LAP-MALDI sample preparation and analysis workflow.

Each species produced a distinctive lipid profile in the *m/z* 600–1100 range, reflecting the unique lipidomes of *C. albicans* and *S. cerevisiae.* High-resolution MS and MS/MS analyses allowed unambiguous identification of key phospholipids including PCs, PEs, and PIs, as well as neutral lipids such as DAGs and TAGs. The identification of [PC(34:3) + K]^+^ at *m/z* 794.51 as well as [PC(32:2) + Na]^+^ at *m/z* 752.52, was supported by characteristic MS/MS fragmentation patterns. Specifically, neutral losses of TMA (~ 59 Da) and the PC headgroup (~ 183 Da) are diagnostic for PCs and are not typically observed in isobaric lipid classes such as ceramide phosphates (CerP), PIs, or PEs. Therefore, although alternative assignments such as PE(37:3) and PE(35:2) are theoretically plausible based on exact mass, their fragmentation behaviour and relative abundance in yeast lipidomes render them unlikely. These identifications are consistent with established fungal lipid biochemistry and underscore the resolving power of MS/MS on high-performing hybrid instruments such as Q-TOF mass spectrometers for distinguishing isobaric lipid species.

The lipidomic distinctions observed are consistent with well-documented differences in fungal membrane composition. *C. albicans* possesses a complex lipidome enriched in phospholipids (PC, PE, PI, PS) and sphingolipids such as inositolphosphorylceramide (IPC) and ceramide phosphates (CerP) which play important roles in virulence, stress response, and antifungal resistance [34–36]. Disruption in lipid biosynthesis, particularly PE and sphingolipids, has been shown to impair pathogenicity and alter drug susceptibility [34, 36]. Importantly, PC(34:3) and other PCs as identified in this study for *C. albicans* have also been amongst the main panels of phospholipids identified in a previous ESI MS/MS study [35].

In contrast, *S. cerevisiae* presents with a more defined lipidome composed of glycerophospholipids, ergosterol, and storage lipids (TAGs, SEs), with fatty acid compositions tuned to regulate membrane fluidity [37–39]. These biochemical hallmarks offer rich discriminatory power for fungal typing and may also provide insights into physiological states or drug resistance.

Traditional clinical MS biotyping instruments, largely based on solid MALDI and axial TOF mass analysers, are optimised for detecting ribosomal and other abundant proteins in the *m/z* 2,000–12,000 range and typically exclude lipids due to detector saturation effects at the lower *m/z* values where abundant MALDI matrix clusters appear in the MALDI mass spectra. In contrast, LAP-MALDI mitigates these limitations. The use of a liquid matrix significantly reduces the presence of high-intensity matrix cluster ions, enabling the clear detection of lipid ions. Furthermore, the orthogonal geometry of Q-TOF mass analysers allows more selective transmission of analyte ions while minimising matrix-induced detector saturation, thereby improving both sensitivity and resolution for lipid analysis. These instrumental advantages facilitate the simultaneous recording of lipid and protein profiles and therefore the seamless integration of lipid profiling into the MS-based biotyping workflows, a significant advancement over current clinical MALDI biotyping systems.

While lipid MS profiles should therefore enable robust species-level differentiation at a similar level as previously shown for bacteria, it is likely that fungal identification based on lipid MS profiling alone may not universally resolve all fungal species or strains. The ability of LAP-MALDI MS to simultaneously detect multiply charged peptide and protein ions adds a powerful complementary dimension. Following C18 ZipTip-based clean-up, distinct protein peaks were observed in both *C. albicans* and *S. cerevisiae*. In *C. albicans*, the mature white colony protein WHS11 was confidently identified with 100% sequence coverage, while in *S. cerevisiae*, dominant signals corresponding to glyceraldehyde-3-phosphate dehydrogenase 3 (TDH3) and the 12-kDa heat shock protein (HSP12) were identified. The latter was also detected in its intact mature form (i.e. 100% sequence coverage). The observed protein masses reflected common post-translational modifications in fungi, including N-terminal methionine cleavage and serine acetylation, consistent with well-known protein maturation mechanisms [40, 41]. The detection of HSP12 with this post-translational modification has been reported in previous studies [24].

The *WH11* gene plays a critical role in regulating the morphological state during white-opaque switching, a phenotypic transition intimately linked to mating competence, immune evasion, and virulence [42]. *WH11* is predominantly expressed in white-phase cells and is markedly upregulated during temperature-induced mass conversion from opaque to white colonies, a process typically triggered by incubation at 37 °C, mimicking host body temperature [42]. Its expression is activated at the second cell doubling during this transition, marking the phenotypic switch [43]. As samples were harvested at this time point in this study, the presence of WHS11 protein can be expected.

TDH3 is one of the most abundantly expressed proteins in *S. cerevisiae*, particularly during the exponential growth phase. As the predominant isoform of GAPDH in yeast, TDH3 accounts for up to 60% of total enzymatic activity and plays a pivotal role in glycolysis and energy production when cells are proliferating under glucose-rich conditions [44, 45]. Its expression is tightly regulated by metabolic state, peaking during the logarithmic phase and declining during the diauxic shift or stationary phase. During these latter stages, other GAPDH isoforms such as TDH1 increase in expression, reflecting an adaptive reprogramming of glycolytic activity. Proteomic analyses estimate TDH3 expression at ~ 12.7 million molecules per cell at peak, underscoring its metabolic significance and its utility as a physiological marker in *S. cerevisiae* [46].

The high abundance of HSP12 observed under optimal growth conditions in *S. cerevisiae* is consistent with its known role in diverse stress response pathways. HSP12 is upregulated during osmotic and oxidative stress, and in early stationary phase. Its expression under non-stressful conditions suggests a constitutive protective function in stabilising membranes and proteins, preventing aggregation, and preserving cellular integrity [47]. Rather than being solely reactive, HSP12 likely contributes proactively to cellular preparedness, ensuring rapid adaptation to environmental changes. This constitutive expression supports its role as a general stress-response protein, potentially explaining its elevated levels even in the absence of acute external stimuli.

The ability to detect both lipids and proteins using LAP-MALDI MS offers several clinical advantages. Firstly, it enhances diagnostic specificity and may facilitate strain-level resolution and resistance profiling, particularly relevant in polymicrobial infections or for tracking resistant fungal lineages. The MS/MS capabilities which are currently excluded from MALDI-TOF biotyping platforms, enable sequence-based identification of proteoforms and structural elucidation of lipid species, further augmenting the analytical power of LAP-MALDI in characterising important species and strains of microbial pathogens. In general, high-throughput LAP-MALDI workflows could readily be developed for fungal biotyping, particularly for lipid profiling, which requires minimal sample processing and is therefore well suited to batch analysis. Automated routines could be implemented to select peaks of interest from MS profiles and lead to targeted MS/MS acquisition, enabling fully automated, high-information workflows based on MS profiles of species-specific biomolecules as well as optional sequence-specific characterisation by MS/MS analysis of both lipids and proteins.

In summary, LAP-MALDI MS enables rapid profiling of fungal pathogens, offering a significant advancement over existing MALDI-based biotyping systems. Through a streamlined ethanol extraction and optional C18 ZipTip clean-up, we achieved rapid mass spectral detection of species-specific lipid signatures and multiply charged proteins directly from cultured colonies. Its ability to simultaneously detect lipid and protein profiles ultimately enhances diagnostic confidence in fungal biotyping based on conventional MS profiling analysis. The additional ability to perform top-down proteoform identification highlights the extended analytical power of LAP-MALDI MS for detailed fungal characterisation and lays the foundation for next-generation fungal identification tools. Future work will focus on expanding the fungal species panel, refining lipid/protein biomarkers, and standardising sample preparation, specifically by removing the ZipTip clean-up step, thus supporting cost-effective clinical implementation and high sample throughput.

## Data Availability

Data supporting the results reported in this paper are openly available from the University of Reading Research Data Archive at 10.17864/1947.001426.
