# Correction to: Speciation analysis of fungi by liquid atmospheric pressure MALDI mass spectrometry

**DOI:** 10.1007/s00216-025-06220-4

**Published:** 2025-11-27

**Authors:** Lily R. Adair, Ian M. Jones, Rainer Cramer

**Affiliations:** 1 Department of Chemistry, School of Chemistry, Food and Pharmacy, University of Reading, Reading, RG6 6DX UK; 2 School of Biological Sciences, University of Reading, Reading, RG6 6AJ UK

**Correction to: Analytical and Bioanalytical Chemistry**

10.1007/s00216-025-06094-6

In the original version of the manuscript, the inset of Figure 2A was incorrectly labelled. While the 10^+^ ion signal is correctly labelled, the 9^+^ and 8^+^ ions were mislabelled as the 8^+^ and 7^+^ ion peaks, respectively, and the 7^+^ ion peak label was omitted. This labelling error has been corrected in the revised figure. The authors apologise for this oversight.


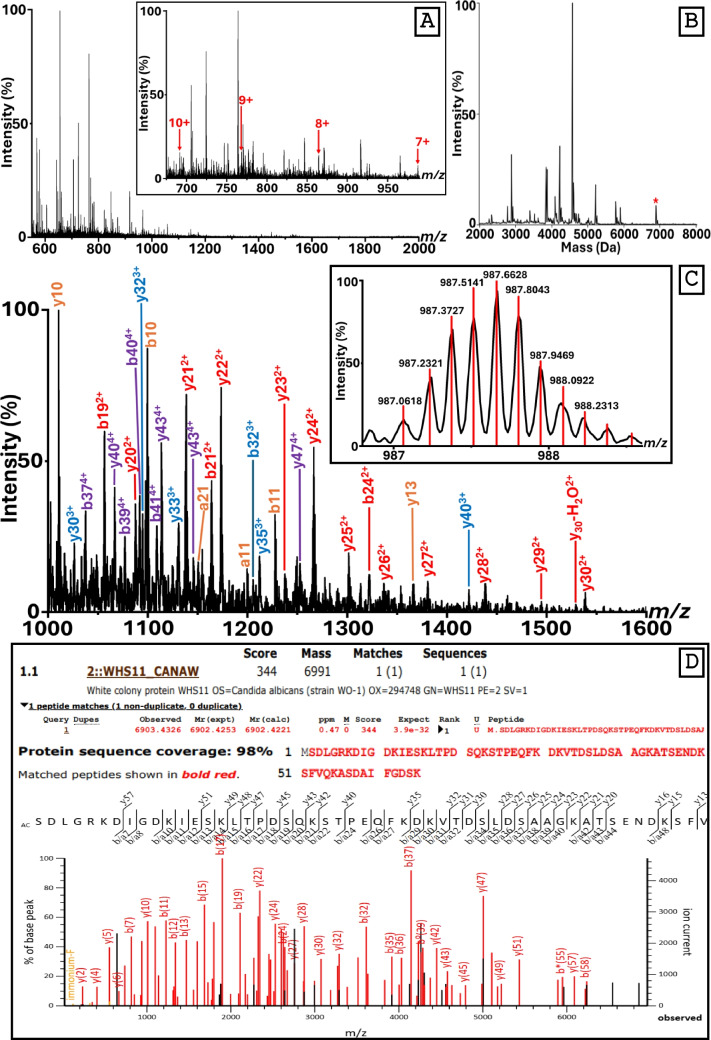


The original article has been corrected.

